# Lost in translation? The dilemma of alignment within participatory technology developments

**DOI:** 10.1007/s10202-012-0118-1

**Published:** 2012-11-13

**Authors:** Diego Compagna

**Affiliations:** Faculty of Social Sciences, University of Duisburg-Essen, Lotharstr. 65, 47057 Duisburg, Germany

## Abstract

As an instrument for participatory technology development, Scenario-Based Design offers significant potential for an early inclusion of future users. Over the course of a 3-year research project, this method was examined as a procedure for participatory technology development. Methods and instruments aimed at achieving a potential user’s participation, and the resulting cooperation of heterogeneous social groups can be seen as translation tools. Their purpose is to act as translators between different social fields and the specific knowledge associated with them. These translation capabilities and participatory methods should result in the best possible convergence of different orientations and purposes. In this paper, attempting to achieve the best possible convergence is described as a dilemma of alignment. Several approaches will be used to describe the dynamic of the alignment dilemma within the above-mentioned project. The reconstruction follows one question that is proposed as a heuristic pattern to meet the requirements of an accurate analysis of holistic participatory methods: Who or what has to adjust to whom or what, why, when, and in which way? The main conclusions include the finding that the alignment dilemma is not equally balanced, that the agency of epistemic objects within the process has to be captured, and that it is easy for translation—provided primarily by core instruments of the participatory method used—to begin to overwrite the needs and purposes of one social group with the interests and orientations of another.

## Abstract (de)

Das Szenariobasierte Design stellt ein viel versprechendes Verfahren für partizipative Technikentwicklung dar. Im Rahmen eines dreijährigen Forschungsprojektes kam dieses Verfahren zum Einsatz und es wurde dessen Eignung für die Verwirklichung partizipativer Entwicklungsvorhaben untersucht. Verfahren und Instrumente, die eine Partizipation der künftigen Nutzer ermöglichen sollen, können als „Übersetzungsinstrumente“ wahrgenommen werden. Ihre Leistung besteht darin, zwischen heterogenen sozialen Gruppen und deren je spezifischen Orientierungen und Wissensbeständen eine Brücke zu schlagen. Ganzheitliche partizipative Verfahren sind dabei von einem Angleichungsdilemma gekennzeichnet. Verschiedene Theorieperspektiven werden herangezogen, um darzustellen inwiefern solche Verfahren als ein Angleichungsdilemma charakterisiert werden können. Die Rekonstruktion des oben genannten Projektes erfolgt dabei entlang einer Frage, die zugleich als heuristische Analysefolie zur Untersuchung partizipativer Entwicklungsvorhaben vorgeschlagen wird: Wer oder was muss sich wem oder was, warum, wann und auf welche Weise angleichen? Die Hauptergebnisse der Untersuchung bestehen darin, dass die Angleichungsanteile zwischen den beteiligten Gruppen ungleich verteilt sind, dass die Handlungsträgerschaft von epistemischen Objekten beachtet werden soll und dass die Übersetzung, die maßgeblich durch die Instrumente des gewählten partizipativen Verfahrens geleistet wird, zu einer „Überschreibung“ der Bedürfnisse und Zielsetzungen einer sozialen Gruppe durch die einer anderen führen kann.

## Résumé

En tant qu’instrument pour le développement de la technologie participative, le «Scenario-Based Design» (conception à l’aide d’un scénario) offre un potentiel significatif pour une inclusion tôt des usagers futurs. Durant un projet de recherche de trois ans, cette méthode a été examinée en tant que procédure pour le développement de la technologie participative. Des méthodes et des instruments visant à maximiser la participation potentielle d’un usager ainsi que la coopération entre des groupes sociaux hétérogènes qui en est le résultat peuvent être considérées comme des outils de traduction. Leur but est d’agir en tant que traducteurs entre les différents champs sociaux et les connaissances spécifiques qui y sont associées. Ces capacités de traduction et méthodes participatives devraient donner lieu à la meilleure convergence possible de différentes orientations et buts. Dans cet article, le fait de tenter d’arriver à la meilleure convergence possible est décrit comme un dilemme d’alignement. Plusieurs approches seront utilisées pour décrire la dynamique du dilemme d’alignement dans le projet décrit ci-dessus. La reconstruction suit une question qui est proposée comme un modèle heuristique avec lequel répondre aux pré-requis d’une analyse correcte des méthodes participatives holistiques: Qu’est-ce qui (personne ou chose) doit s’ajuster à qui ou à quoi, pourquoi, quand, et de quelle manière? Parmi les conclusions centrales figurent le fait de trouver que le dilemme d’alignement n’est pas équilibré de manière égale, que l’intermédiaire des objets épistémiques dans le processus doit être capturé, et que la traduction peut facilement—facilitée surtout par les instruments fondamentaux de la méthode participative utilisée—commencer à écraser les besoins et les buts d’un groupe social par les intérêts et orientations d’un autre.

## Introduction

In a very broad sense, every method that is used to achieve participatory technology assessment (pTA) or development is a translation tool. The goal is the successful translation of specific knowledge from one social field into another. In this light, the need for effective methods of participation only arises in a crucial way in functionally differentiated societies. Highly specialized social fields and the very specific knowledge related to them (e.g., different fields of technological developments) have to be translated into everyday user-knowledge and/or the other way around (Fung 2006). Especially in technology assessment, one observes asymmetry between specialists’ highly specific knowledge of technological developments on the one hand and the somehow “unspecific” everyday knowledge of potential users on the other (Chilvers 2008; Eijndhoven and Est 2002). One challenging problem that arises from this disparity in knowledge can be seen in the need to fill the user’s lack of knowledge and, by doing so, to change him or her into a non-professional or lay-person expert (Felt and Fochler 2010; Braun and Schultz 2010).

This paper primarily deals with a slightly different situation that serves as a typical setting for participatory technology development (pTD). In cases such as these, the user’s social field is usually a clearly defined, specialized area (Törpel et al. 2009). Therefore, the slight but significant difference between pTA and pTD is that in pTD the user groups also participate as experts in their field. The above-mentioned asymmetry in pTA is made more symmetrical in pTD. In an ideal pTD situation, the user and the technicians are both characterized by a lack of knowledge regarding the opposite group. In this setting, the participatory method should be able to accomplish the ambitious goal of creating equal translations that are balanced in both directions at the same time. The main reason for these differences is the result of the settings in which pTD is usually conducted. Due to the fact that pTD primarily focuses on the development of a specific technological tool to be used in a clearly defined field, the future user is also the expert in the field of intended development. In contrast to common pTA situations, the user is not a more or less randomly chosen member of a vast user group but rather a highly specialized professional actor in the field of intended development. In both participatory methods, pTA and pTD, the challenge lies in realizing a successful translation. However, the demands regarding the participatory method as a tool for translation are higher in pTD because it requires a balanced bidirectional translation between two highly specialized areas of knowledge.

The ideal pTD method for fruitful exchange between the involved parties is capable of transferring heterogeneous contents among them without turning them into experts in one another’s area of expertise. This is why ‘translation’ is an appropriate metaphor as well as concept of the Actor-Network-Theory (Callon 1986, 1991) for expressing the main objectives of pTD methods: The process is similar to that of a translator enabling a discussion between individuals who speak different languages. The benefit lies in making such a discussion possible without the participants needing to invest time or other resources to learn each other’s (foreign) languages. Participatory methods serve a similarly beneficial service within functionally differentiated societies. Beyond the previously mentioned similarity, the situation is more complex in pTD. First and foremost, the constraints of the technology have to be taken into account as well as the involved groups’ different orientations and goals. There is a very high probability that one group’s orientation and goals will need to be adjusted in terms of another group or in terms of the restricted range of capabilities of the technology (Schreuer et al. 2010; Raven et al. 2008). For this reason, the instruments that are used to achieve these goals are very specific: They are responsible for whether and how relevant information is transferred from one group to another. Again, the metaphor comparing pTD with the work of a translator helps underline a crucial point: Since the involved social groups do not understand each other, it is crucial that the pTD method’s instruments are impartial and detached from the interests of both the users and the developers.

In analyzing a case study with the aim of realizing a pTD, I want to show that, by providing an adequate bidirectional translation among heterogeneous social groups, the instruments used to enable exchange play a crucial role insofar as every participating group has to adjust according to the instruments themselves. Since exchange takes place primarily through the implemented pTD method’s instruments, it is important to analyze their roles in the participation process carefully. In the end, participating user groups have to adjust their expectations, needs, and ideas primarily according to the instruments that represent participating developer groups’ expectations, capabilities, and resources—and vice versa. Therefore, the following analysis could be summarized as the answer to the question: *Who or what has to adjust to whom or what, why, when, and in which way?*

To answer to this paper’s stated central research question, I will focus on the role of translation tools, that is, the pTD method’s instruments. The main theoretical framework I have selected is the Actor-Network-Theory (ANT), because it allows objects to be conceptualized as comparable to human actors, an important feature of this theory in regard to one of my main assumptions: the relevance of participation instruments as translation tools. At the same time, I would like to emphasize the bargaining process among social groups and highlight how instruments are used (either intentionally or unintentionally) in a manipulative way. Even if the instruments have a certain agency of their own and produce unintended effects, to a certain extent, this can still be traced back to the social groups and their orientations and goals. On these grounds, I will confine ANT to two approaches that focus on interactions between social actors and groups in heterogeneous social groups’ cooperation: The Social Constructivism of Technology (SCOT) and the concept of Boundary Objects (BO). Finally, I will point out some limitations associated with ANT and suggest how they could be improved by being combined with SCOT.

## Case study: the implementation of service robots in a care facility for the elderly

The case study that will be used to analyze the peculiar “dilemma of alignment” (Ornetzeder 2010: 44) within pTD was a research project lasting 3 years, starting in October 2008. The method of participation in this recent research project was Scenario-based Design (SBD). This approach systematically implements scenarios as core instruments for accomplishing user-oriented, participatory development of new technology (Rosson and Carroll 2003). This project’s primary objective was to successfully include relevant user groups in the development process of two different service robots. Due to the fact that this project’s background was a shift in demographics, the robots came into use in a care facility for the elderly.

There were a total of three main user groups and four developing groups: The residents of the facility (elderly in need for care), the care workers, and the management each formed one user group. The developing groups included two different kinds of engineers (research-oriented and profit-oriented, depending on organizational background), designers (in charge of conceptualizing the scenarios and measuring usability benchmarks), and social scientists (responsible for conducting a requirement analysis with the aim of identifying user needs and potential useful scenarios).

For methodological purposes, it is important to point out that after the previously mentioned task, the social scientists’ main responsibility was to examine knowledge transfer throughout the process. This put the social scientists in the challenging role of accomplishing a task within the process and, at the same time, analyzing a process in which they were involved (especially in the beginning). Despite the fact that this was clear from the beginning and was subject to constant critical self-reflection, most of the “constructive” input was completed in the project’s first year. So in the two following years, the social scientists were able to focus on the intended pTD research. Nevertheless, this is a source of bias to bear in mind (quite typical for this kind of project), especially since the author was an active member of the social scientists’ team.

**Fig. 1:**
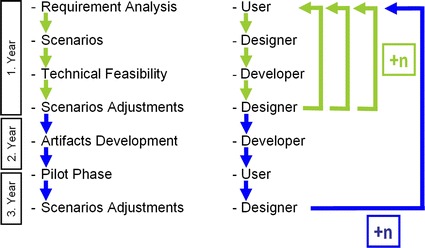
Knowledge-transfer-loop in pTD adopting the SBD (*left column* most important steps, *right column* most involved group in each step)

Figure 1 describes the whole process over the project’s 3-year duration—how SBD was implemented to find a user-oriented way of further developing robots to be used in a care facility. There are two clear feedback loops that I would like to refer to as knowledge transfer loops. Both looping sections are characterized by attributes of oscillation and iteration: oscillating between user and developer and repeating this oscillation until all involved groups were satisfied with the outcome (Mack 1995).

Within the first looping section, the outcomes are the scenarios themselves. Within the second looping section, the robots’ concrete operations in pilot tests (shaped according to previously determined scenarios) generate output simply by testing artifacts’ functionality and gathering user feedback. The SBD’s scenarios are drawn up by putting the planned implementation of artifacts into a very vivid, narrative sequence of sketches, usually similar to a comic strip or a storyboard used for movies. One positive outcome of this descriptive and detailed elaboration on the planned scenarios is the opportunity to gain valid feedback from all involved social groups (Erickson 1995). Even people who have never interacted with a robot can imagine the planned implementations when they are confronted with the scenario sketches and asked for an assessment of them.

Scenario selection is determined largely by two factors: First, they result from requirement analysis, and second, they are selected according to technical feasibility. The shape of the selected scenarios is again the result of two factors: First, they are based on user groups’ needs and notions, and second, feasibility according to the developers’ available resources. These two stages are not completely separate; nevertheless, they can be identified as two consecutive steps in the first looping section.

After identifying a total of 12 possible scenarios that emerged from the requirement analysis conducted by the social scientists in the care facility and discussing them with the robots’ engineers, six of the scenarios had to be omitted due to general technical feasibility. The remaining six scenarios also had to be adjusted to accommodate the involved research institutes’ and companies’ limited resources.

The next step consisted in shaping the scenarios: This step required several iterating loops between the users and the engineers. After showing the six remaining and adjusted scenarios to the user groups in the facility, two scenarios were omitted by the users because after they had been adjusted by the developers, they no longer served the users’ needs and notions. The remaining four scenarios had to be slightly adjusted based on user feedback and were then discussed again with the engineers. This adjustment-looping process was repeated until every party was satisfied—at this point, one can assume that the remaining four adjusted scenarios are both socially desirable and technically feasible. Table 1 comprises the four scenarios that were later tested in pilot applications, including a brief description.

**Table 1:** Four selected scenarios to be accomplished with service robots in a stationary care facility for the elderly

Transport-scenario	This scenario deals with routine logistical tasks that occur in care facilities—the transport of food trays, medication, laundry, waste, and mail. The automated transport of these items should relieve staff members of time-consuming and cumbersome work that is not and should not be their primary focus
Night-duty-emergency-scenario	This scenario involves providing assistance to care workers during the night shift. Goals of this scenario include increased patrol-coverage, decreased reaction time in emergency situations, providing contextual information in emergencies, serving as a first-aid station, and finally, serving as a two-way communication station
Drink-catering-scenario	This scenario’s main task is providing residents with beverages. The nurse orders the robot to prepare various cans, mugs, etc. The robot should be able to distribute drinks autonomously and to know how much it has given to each resident. Finally, a summary of the drinks served by the robot should be provided
Activity scenario	The goals of this scenario include providing support during occupational therapy and providing a rich database of games, songs, poems, etc. Tending to everyday care tasks leaves only a short amount of time to provide entertaining activities for the elderly or even to encourage them to interact with other residents

The scenarios can be described as epistemic objects: They are both imaginary and tangible and, through the process of adjustment, they increasingly become the products of specific knowledge combined from the various social areas involved. They are able to serve as instruments for translation because of their vividness and their openness to interpretation. This means scenarios are both relatively detailed descriptions of a possible future and, at the same time, vague or open to new interpretations. This interplay is characteristic of scenarios used in SBD as well as of epistemic objects in general (Nardi 1995). Epistemic objects are characterized by their need for interpretation, the need to give them meaning when dealing/working with them, and the fact that they are usually “made” in social contexts characterized by a high density of knowledge (Heintz 2000: 110ff; Latour 1997; Rheinberger 1997).

The impact of the user’s contribution is very high in the first looping section and relatively low in the second. In the pilot phase, all the relevant decisions have already been made, and users’ influence is reduced to feedback regarding the functionality of concrete developments. The interpretative flexibility of the artifacts in terms of the way they will be shaped and introduced into the environment is significantly higher in the first looping section than in the second (Bijker 1997: 73ff). This is the reason for emphasizing the scenarios as epistemic objects and therefore the outcome of the bargaining process that takes place primarily within the first looping section of the implemented pTD. Within this section, the entanglement between the involved social groups and the scenarios became a showcase for the dilemma of alignment within the pTD process, which I would like to characterize as involving translation between heterogeneous and partially incommensurable areas of knowledge. As epistemic objects, the scenarios play crucial roles because they take on the challenging task of translating between highly heterogeneous areas of knowledge. In light of the fact that the entanglement between social groups is mediated by scenarios, it is of paramount importance to focus on the scenarios as epistemic objects.

## Lost in translation: the entanglement between epistemic objects and social groups

Scenarios are generally seen as powerful instruments for the successful development of technology (Konrad 2004). They represent clear images for the planned implementation of “new”, developed artifacts into the social contexts for which they are designed. In fact, there is a significant difference between scenarios and visions: Visions are far vaguer and less precise pictures of a possible future. They can therefore capture a wider spectrum of societal impact, enabling general assessment on a broad scale—whereas scenarios are quite detailed, mostly narrative orchestrations that imagine the impact on a clearly defined social situation if new technology were implemented as planned (Grunwald 2010: 104ff; Schulz-Schaeffer 2012).

According to the descriptions in the literature (e.g., Carroll 1995a; Rosson and Carroll 2003), SBD is characterized as an instrument for participatory technology development that seems to offer significant potential for early inclusion of future users. The main features referred to in this regard are the clarity with which scenarios could be presented and discussed among participating groups of developers and users, as well as the iterative process of adjustment. Furthermore, the literature emphasized the pilot phase’s application procedures as important features in ensuring an ideal exchange among users, designers, and developers (Erickson 1995; Mack 1995). The interplay of these features is expected to result in an optimal balance with regard to social desideratum and technical feasibility. This procedure was nearly completed in the previously mentioned 3-year research project. The case study’s analysis revealed a significant breaking point that affected the procedure’s ambitious aims: The scenarios’ agency as epistemic objects created an imbalance in favor of the developers’ interests and orientations. Since the exchange was realized primarily through the pTD method’s instruments, it is important to analyze their role in the process of participation carefully. The salient question is now: How were the scenarios converted into instruments that overrode the other groups’ interests?

The process that leads to the outcome of one specific scenario is a suitable platform for discussing the entanglement among the scenarios as epistemic objects and the social groups involved. As follows from the descriptions in the last section, the whole process seems to be guided by two main social groups, the engineers and the users. Another group involved in the process served as mediators by transforming user needs according to potential scenarios and making adjustments based on the developers’ feasibility feedback. Table 2 lists the participating groups and assigns them to the three different categories: user, mediator, developer.

**Table 2:** Participating social groups and their roles in the pTD process

(*U*-*A*) Facility residents (elderly in need of care)	User
(*U*-*B*) Facility care workers	User
(*U*-C) Facility management	User
(*M*-*A*) Social scientists (responsible for conducting requirement analysis with the goal of identifying user needs and potential useful scenarios)	Mediator
(*M*-*B*) Designers (in charge of conceptualizing the scenarios and measuring usability benchmarks)	Mediator
(*D*-*A*) Engineers (research-oriented, based on organizational background)	Developer
(*D*-*B*) Engineers (profit-oriented, based on organizational background)	Developer

On the one hand, the scenarios are the products of the mediating groups. On the other, they are products of all the groups involved, functioning as translation tools among them. Though they appear to be mere instruments without their own agency, the scenarios impact the whole situation considerably. The four scenarios that were finally chosen included one so-called “activity scenario” (see Table 1). This scenario was problematic from the beginning of the whole process: Although the conducted requirement analysis showed it to be one-sided, it clearly lacked a necessary condition. Some of the care workers could see the value in trying out such a scenario, but the most important user group, the residents of the care facility, generally refused such an intense interaction with the robots.

The bargaining process’s dynamic was of utmost interest. The agreements made among the different groups, especially from the developers’ point of view, are crucial in understanding how this scenario made it through the whole process and was tested in at least one of the pilot stages. The groups most deeply involved in this bargaining process were the social scientists, who conducted the requirement analysis (*M*-*A*); the designers, who created the scenarios sketches (*M*-*B*); and the engineers of the service robot that was manufactured in a research facility for assistive robotics (*D*-*A*). This last group was interested in an entertainment scenario because their service robot was originally designed and developed for this type of application. By examining these, one can identify a typical case of path dependency. The robots’ engineers were obviously interested in further developing the artifact’s entertainment capabilities and testing them in real, everyday environments. The designers had a more or less neutral position. However, they were fascinated by the challenge of designing a proper user-interface for such an application. In contrast, the social scientists were very critical and their tendency was to dismiss this scenario entirely because it was only partly accepted by the elderly residents. The first observation to keep in mind is that at this specific stage of the scenario-building process, the user groups were absent, or rather they were represented by the social scientists, the designers, and those scenarios that had been selected and depicted at that point.

The engineers presented a very convincing argument in favor to the activity scenario. They pointed out that the elderly residents might be predisposed to rejecting certain scenarios because it is difficult for them to imagine what interacting with a robot would be like. Therefore, an activity scenario could be a useful test. The social scientists expanded this explanation and construed it according to their own relevance system (i.e., horizon of meaning). By referring to Popper’s falsification premise, they claimed that it is important to add a scenario to the developing process from which the requirement is missing, because this is the only way to find out scientifically if requirement-oriented development (and pTD in general) is important (Popper 2005; Wellmer 1967).

It is instructive to describe this notion from a SCOT point of view before describing it with ANT concepts. According to the basic principles of SCOT, the process mentioned could be described as problem re-definition aimed at providing successful closure in terms of development and the relevant social groups involved in the process. In the famous article that introduced this concept, the authors Pinch and Bijker (1999) point out that the phenomenon of problem re-definition is crucial for providing closure in heterogeneous social groups with different—and usually conflicting—interests and orientations. One specific form of “new” technology that represents a solution for some of the involved groups could achieve successful closure if other relevant social groups also see the new artifact as a solution according to their specific interests and orientations (Pinch and Bijker 1999: 44ff).

However, a closer look reveals that the problem was not re-defined, but rather the outcome. The SCOT approach actually places strong emphasis on the social aspect. One frequent criticism of SCOT is that the social aspect holds too much weight, that by proposing a theory dissociated from technological determinism, SCOT ended up providing the exact opposite—an explanation of technological developments based on sociological determinism (Hennen 1992: 41f; Schulz-Schaeffer 2000: 26ff; MacKenzie and Wajcman 2010: xiv). Especially in the first period, when SCOT presented itself as a new paradigm for sociological analysis of technology, the argumentation tended to treat the social aspect as changeless and fixed. In turn, technology had to be very mutable and was adjusted to the fixed needs of the social aspect. The burden of developing technology successfully lay solely on the social aspect and the reconciliation of various orientations and problems of the relevant social groups involved. In fact, what is proposed as problem re-definition is solution re-definition. What was originally presumed to be the solution to a problem was re-defined by technology presenting a new solution to the same problem that could be now solved in a new, previously disregarded way (Pinch and Bijker 1999: 44ff). A single social group’s goal and its related problems remain the same: What can change is the way this social group is able to imagine a (new) solution to the (same) problem.

By advocating the activity scenario, the engineers forced the social scientists to respond to the scenario itself. In order to do so, the social scientists had to come up with a completely new goal and related problems to solve, for which the activity scenario could be seen as a solution. This is a completely different situation than described by the “classical version” of SCOT because the problem for which the activity scenario could be seen as a solution did not exist beforehand. The social scientist’s motivation could be seen as the effect of the necessity to provide a strong link to the scenario as a relevant part of the developing network, or pTD-network.

Before further unfolding this line of argumentation toward ANT, I would like to discuss another dominant perspective on heterogeneous cooperation by characterizing the scenarios as boundary objects (BOs). In the late 1980s, when Star and Griesemer (1989) presented the BO concept to describe successful cooperation between heterogeneous social groups, this concept was a critical reaction against the up-and-coming ANT (Strübing 1997: 374f). For this reason, it was very important for them to emphasize that the meaning of a BO remained different for each participating social group over the whole process of cooperation and that, in spite this, cooperation could still be very successful.

“Boundary objects are objects which are both plastic enough to adapt to local needs and the constraints of the several parties employing them, yet robust enough to maintain a common identity across sites. They are weakly structured in common use and become strongly structured in individual site use. These objects may be abstract or concrete. They have different meanings in different social worlds but their structure is common enough to more than one world to make them recognizable, a means of translation. The creation and management of boundary objects is a key process in developing and maintaining coherence across intersecting social worlds.” (Star and Griesemer 1989: 393)

Although the premise of static denotation over the course of the whole process was broken down in subsequent publications by Star (e.g., together with Bowker), the crucial assumption remained that meaning was different for each social group (Bowker and Star 1999: 254, 296ff). In contrast, ANT argues that successful cooperation is only possible if the cooperation becomes a network in which each participant or actant (these are social groups, individuals, as well as objects) is adjusted to the others, especially to those actants in focal positions within the network. These are described as “Obligatory Passage Points” (OPP) when it is imperative that all the network’s other actants relate to them (Callon 1986; Law 1999). When comparing a BO approach with ANT by adapting both to an empirical case study, one will usually find that what can be identified as a BO, is, from an ANT perspective, most likely an OPP. One fundamental difference remains that makes it more appropriate to describe the scenarios that were used in the mentioned case study as OPPs than BOs. The main reason for this is linked to the way the process of relating occurred: The social scientists’ primarily relation to the activity scenario could not help form a stable cooperation unless they changed it in a positive way. The social scientists had to find a way to relate to this scenario—at whatever cost—to keep the cooperation running smoothly and to avoid the risk of failure, or at least without losing one of the developer groups (*D*-*A*).

The agency of the activity scenario is related to its attributes as an OPP or, in other words, as a BO with the ulterior attribute of categorical necessity, which leads to an object-related agency. The activity scenario attained agency simply through participating social groups’ appreciation of it as a crucial piece in the network or cooperation. It became a BO by those means, even though its demanding character, which one may describe as a kind of object-related agency, is somehow missing in BO. Therefore, ANT provides a more appropriate assessment of the situation from this point on, if only because ANT emphasizes that both people and objects have equal influence in establishing a stable socio-technical system (network, cooperation, or whatever denomination for societal context that basically includes objects/technique).

ANT turns out to be highly appropriate in capturing and describing the crucial elements of this exemplary entanglement. The scenarios are central to the bargaining process. They are strategic and definite OPPs for pTD purposes: Each social group (including the user groups and the artifacts) had to align itself to the selected scenarios. Of course, one important strength of the scenarios is their flexibility, openness, and ability to be modified. But the scenarios’ general orientation is the main issue at stake in the previously mentioned bargaining process. The question is whether or not the activity scenario should be included. If the decision is “yes,” the next question for every participant is to find out how to align him- or herself to it.

## Discussion: the dilemma of alignment in participatory technology development

In each individual case, the way alignment occurs can be influenced either by the relevant social groups involved, the technology, or epistemic objects that emerge (they may be, in part, explicitly participation-related instruments) when a participatory method is used. Though these differentiations are crucial analytical classifications, within the process of pTD, they are entangled and vague. The following analysis will emphasize analysis of the intrinsic enmeshment of these three main categories (social groups, e.g., engineers, designers, users, technology, and epistemic objects) by first dividing them and then putting them back together; a procedure that ANT would describe as re-assembling the black box (Latour 2005).

In terms of ANT, it is possible to capture the dynamic of the scenarios itself, since a scenario (as a program) overrides the results of the requirement analysis (anti-program) by “translating” or “overwriting” the social scientist’s argumentation (Latour 1991). At this stage, the translation process takes place between the activity scenario and the social scientist. This becomes evident if one takes into account the fact that the argumentation strategy that the social scientist chooses to deal with the activity scenario is related to his or her general perspective (which is a scientific one). The social scientists did not simply copy the engineers’ argumentation, but rather came up with their own strategies for adjusting and connecting with the activity scenario by referring to an argument related to methodology (i.e., the falsification premise of Popper’s critical epistemology). It is primarily for this reason that the ANT perspective takes preference over other competing approaches: ANT is able to observe and capture the structuring or networking process without loosing track of the relevance of objects—in regard to the selected pTD example, the scenarios’ contribution as epistemic objects especially should be included in the analysis.

**Fig. 2:**
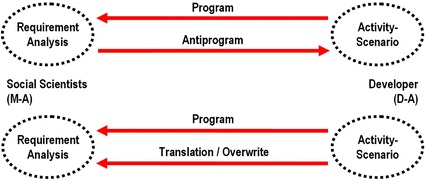
Programming and translating/overwriting within the discussed example

I would like to return to the metaphor of translation and define it in a more precise way by relating it to ANT terminology. Figure 2 depicts its main argumentation, focusing on the dispute between the social scientists and the developer. The term “translation” is used very differently here than in the previous description of pTD as a translation process. The way it was introduced by Callon in his seminal contribution to ANT “Elements of a sociology of translation” (1986)—and elaborated further later on (1991)—the term describes a relation between actants that is characterized by inequality and that leads to a network structure based (at least in part) on power conditions. The dynamic of the given example could also be described in terms of a translation process: Different orientations or interpretations as to how the network should function or be structured is overwritten (i.e., translated) by the program. In this regard, the program of the activity scenario is stronger than the program of the requirement analysis’s findings, which, in retrospective reconstruction of the networking process, becomes an anti-program (Latour 1991; Knorr-Cetina 1995: 117f). The strength of the activity scenario lays in the high grade of innovation which was able to overrule the user’s assessment due to their reputedly incapability of imagination.

“‘*A* translates *B*’. To say this is to say that *A* defines *B*. It does not matter whether *B* is human or non-human, a collectivity or an individual. Neither does it say anything about *B*’s status as an actor. *B* might be endowed with interests, projects, desires, strategies, reflexes, or afterthoughts. The decision is *A*’s—though this does not mean that *A* has total freedom. For how *A* acts depends on past translations. These may influence what follows to the point of determining them. […] The notion of translation thus implies definition […], it makes little sense to speak of translation ‘in general’. We have to define the medium, the material into which it is inscribed: roundtable discussions, public declarations, texts, technical objects, embodied skills, currencies—the possibilities are endless. Nevertheless the elementary operation of translation is triangular: it involves a translator, something that is translated, and a medium in which that translation is inscribed.” (Callon 1991: 143)

According to this definition, the activity scenario itself takes the place of the translator, the findings of the requirement analysis were translated (overwritten) by it, and the social scientists act as the medium. This theoretical perspective is linked to another aspect that should be emphasized: As previously mentioned, translation in pTD is bidirectional to such a degree that users take the role of experts as highly skilled as the developers, so the instruments of the pTD method receive the status of OPP (Callon 1986). This means that the cooperating groups have to relate to the translation instruments. In regard to the example: The involved social groups have absolutely no choice: Each group has to align itself to the scenarios, otherwise they can no longer participate in the collective process of shaping new developments and would be rendered “speechless.” Metaphorically speaking, the only language every group can understand is the “scenario-language.” Upon agreeing on a certain pTD method, every group also agreed to which “language” they would use to speak to each other. Even if the scenarios can be characterized as epistemic objects due to their vague nature and their need for interpretation, within the mentioned case study, they are the only legitimate tools for translation—and for this very reason, they acquire quality of very strong overwriting-tools.

In the last section, I highlighted the relevance of epistemic objects in pTD. On the one hand, epistemic objects are the result of a translation process, and on the other, they can play active roles within the translation dynamic. The previously mentioned example was chosen for its appropriateness in emphasizing the critical aspects of the translation of knowledge in pTD. The indicated entanglement between heterogeneous social groups, technology, and epistemic objects takes the shape of an alignment dilemma as soon as the question is raised: “Who or what has to adjust to whom or what, when, why, and in which way?”

As I stated in the opening section of this paper, this question includes the main dimensions of pTD insofar as pTD is characterized primarily as a translation process. From this point of view, this question has a heuristic value. If adapted to the given example, what could in the end appear to be the effect of a strong bias or some sort of leakage within the translation process that leads to the realization of an unneeded and non-required scenario still highly integrated into the pTD-network, the heuristic value becomes visible: Who (the social scientists) or what (the results of the requirement analysis) had to align to whom (interests of the service robots’ engineers) or what (the activity scenario in turn related to the path dependency of the artifact’s developments), when (in the last step of the first scenario-adjusting-looping process), why (to be able to deal with a crucial element of the pTD-network, that is, OPP), and in which way (by finding a new goal/problem related to their own main perspective, for which the scenario could serve as a solution).

Though ANT provides an accurate description of the “who to whom or what” as well as the “what to whom or what-adjustment,” it is not suitable for accurately explaining the “why, when, and in which way” components of the dynamic process. For this reason, I would like to combine ANT with some assumptions of SCOT. The concept of semiotic power has proved especially helpful. It was developed as a supplement to SCOT, with the aim of capturing (in an appropriate way) aspects of hierarchy and domination within technological development processes and the relevant social groups’ corresponding bargaining dynamics (Bijker 1997: 260ff). This concept can be seen as a reaction to Winner’s (1993) demanding criticism of SCOT. Pursuing a similar purpose—to include the effects of power in the analysis—Callon also developed the concept of translation and OPP (Callon 1986: 196f).

In adapting the ANT perspective to the alignment process, one notes the entanglement between objects and social groups. It is important to examine the situation from a broad perspective: This includes the general effects and hidden implications of the artifacts, the specific technology, the environmental setting, as well as the epistemic objects involved. To understand how the scenarios (as epistemic objects) organize and shape the pTD process as a whole, it is also important to choose a theoretical perspective that can describe a bargaining process between human actors and objects. However, theories that emphasize social groups and the bargaining processes that take place between them should be reconsidered because epistemic objects are the results of ongoing social processes: Even if a scenario is able to transcend its original lack of relevance as an object, it is also important to understand the way the interests, orientations, and limited resources of one social group (the engineers) can promote an originally unnecessary application in favor of their service robot developments. One possibility is to emphasize Bijker’s semiotic power approach to examine how one relevant user group (*U*-*A*) was redefined.

“The semiotic and micropolitical aspects of this power conception can be directly linked to the closure and stabilization processes […]. The reaching of closure, whereby the interpretative flexibility of an artifact is diminished and its meaning fixed, can now be interpreted as a first step in constituting semiotic power, resulting from a multitude of micropolitics to fix meanings. […] A technological frame then constrains actions of its members and thus exerts power through the fixity of meanings of, among other elements, artifacts; this is the semiotic aspect of the new power conception. A technological frame also enables its members by providing problem-solving strategies, theories, and testing practices, for example, which forms the micropolitical aspect of power.” (Bijker 1997: 263f)

Even if the above allocation is appropriate with regard to the three main elements of a translation process in terms of ANT, following Bijkers’ notion of semiotic and micropolitical power leads to a different evaluation: Table 3 compares the two different approaches in terms of the three elements of translation. The different focal points of the two approaches are obvious: With ANT, two active elements are epistemic objects (the activity scenario and the requirement analysis’ findings), whereas the medium is a social group (the social scientists). In contrast, with SCOT, the medium is an epistemic object (the activity scenario), and the active parts are played by social groups (one developer and one user group):

**Table 3:** Comparison of ANT and SCOT in regard to the elements of translation

	Translator	Translated	Medium
ANT	Activity scenario	Requirement analysis	Social scientists (*M*-*A*)
SCOT	Engineers (*D*-*A*)	User (*U*-*A*)	Activity scenario

The salient point in this detail of the case study’s whole pTD is the way the robot engineers’ initial argumentation to include the activity scenario in the process had a long-term effect on the structure of the pTD-network, which SCOT’s definition captures more appropriately. Arguing that the elderly’s negative assessment of the human–robot interaction arose from lack of experience excludes this group from the intended user participation. However, in the end, this argumentation sought to bring this group back into the role of users in the second looping process, which consists of carrying out the pilot tests. The “success” of the activity scenarios’ program over the anti-program of the requirement analysis findings leads to an immediate redefinition of one user group’s role. The translation in the top line (ANT) of Table 3 also affected the power dynamic between some of the participating groups, which is represented more adequately in the bottom line (SCOT) of the table.

Still following the guidelines of Bijker’s semiotic power approach, the second notable change in the pTD structure is the social scientists’ previously mentioned necessity to align themselves to the activity scenario. As an epistemic object, the scenario reflects the engineers’ interests and the artifact’s involved path dependency (which of course far exceeds the interests of the engineers involved and must be located on an organizational level). On the other hand, it took on the value of an epistemologically accurate scientific way to deal with the pTD situation. Furthermore, when it eventually materialized and was conducted as a pilot application, it finally became apparent that the hierarchical structure between the participating groups is directly affected by the prior translation process.

Describing the process with ANT proved to be an appropriate way to capture the relevant elements that explain the dynamic and (at least some relevant parts) of the entanglement. However, using SCOT to some extent could improve assessment of the results of a single but crucial bargaining step in terms of the whole process of pTD and the resulting structure in a long-term way.

## Conclusion

In an overall evaluation, the SBD turned out to be a useful participatory method for pTD. The sketched narrative scenarios were generally able to function as translating tools, capable of including users in the development process. However, their agency in combination with diverging orientations of the involved social groups has to be kept in mind. One suggestion to meet such demands was to describe the process of pTD as a bidirectional translation among heterogeneous social groups. Raising the question *Who or what has to adjust to whom or what, why, when, and in which way?* served to characterize it as a dilemma of alignment. Based on a case study that strived for participation of all relevant developer- and user groups, the difficulties of the bidirectional translation were described by adopting some of the ANT’s main assumptions and eventually by combining them with SCOT’s semiotic power approach with the aim to gain further details. The particular significance of the scenarios—which function as the procedure’s central instruments (Carroll 1995b)—is their function as translation tools and, at the same time, their ability to override a participating user’s demands (Nardi 1995). ANT has proved capable of properly capturing the scenarios’ dynamic roles. As core instruments for translating between different social groups, the scenarios have to be such that every group can align itself to them, and at the same time, they must. Therefore, the scenarios can be characterized according to ANT terminology as OPP (Callon 1986).

The main assumptions of this paper can be summarized in three parts: First, the significance of participatory methods is strictly interwoven with the basic structure of actual modern societies and their functional differentiation. Second, their main goal is to provide translations between various highly specialized knowledge from different social fields. The translation is affected by several factors: by the relevant social groups and their positions in relation to one another—which mainly depends on the rate of knowledge-related symmetry that, in turn, depends on the density, that is, degree of specialization, of the social field. It is also affected by the technology itself, its limitations and enabling potentials. Third and finally, the intended translation properties of participatory methods turn out to include an alignment process. This process could be characterized as a dilemma, seeing that—apart from distinct limitations of the involved technology—the way (who and what) the alignment is processed is not determinate but rather contingent and influenced by different factors simultaneously. In the end, the term “translation”—as it was introduced by Callon within the framework of ANT—is expanding in a vital way what was previously used only metaphorically to describe the background and constitution of participatory methods. In the analyzed case study, the scenarios—the core instrument of the implemented pTD method—turned out to be both instruments allowing cooperation among heterogeneous social groups as well as “translators” in terms of ANT (Callon 1986, 1991). It is crucial to point out this double feature of the scenarios: They play both a passive role (as translation instruments) and an active one (as translating entities in the successful consolidation of cooperation—ANT would call it network). As epistemic objects, the scenarios themselves are both the outcome of the intended translation process and, at the same time, active translators with the intrinsic ability to recompile and reconfigure the whole setting in the dynamic process of assembling the intended “new” socio-technical situation.

## References

[CR2] Bijker WE (1997). Of bicycles, bakelites, and bulbs. Toward a theory of sociotechnical change. (2. Aufl.).

[CR3] Bowker GC, Star SL (1999). Sorting things out. Classification and its consequences. (1. Aufl.).

[CR4] Braun K, Schultz S (2010). ‘‘… a certain amount of engineering involved’’. Constructing the public in participatory governance arrangements. Public Underst Sci.

[CR5] Callon M, Law J (1986). Elements of a sociology of translation. Domestication of the Scallops and the Fishermen of St Brieuc Bay. Power, action and belief. A new sociology of knowledge? (1. Aufl.).

[CR6] Callon M, Law J (1991). Techno-economic networks and irreversibility. A sociology of monsters. Essays on power, technology and domination. (1. Aufl.).

[CR1] Carroll JM (1995). Scenario-based design. Envisioning work and technology in systems development. (1. Aufl.).

[CR7] Carroll JM, Ders. (1995). Introduction. The scenario perspective on system development. Scenario-based design. Envisioning work and technology in systems development. (1. Aufl.).

[CR8] Chilvers J (2008). Deliberating competence. Theoretical and practitioner perspectives on effective participatory appraisal practice. Sci Technol Hum Values.

[CR9] Erickson T, Carroll JM (1995). Notes on design practice. Stories and prototypes as catalysts for communication. Scenario-based design. Envisioning work and technology in systems development. (1. Aufl.).

[CR10] Felt U, Fochler M (2010). Machineries for making publics. Inscribing and de-scribing publics in public engagement. Minerva.

[CR11] Fung A (2006). Varieties of participation in complex governance. Public Adm Rev.

[CR12] Grunwald A (2010). Technikfolgenabschätzung. Eine Einführung. (2. Aufl.).

[CR13] Heintz B (2000). Die Innenwelt der Mathematik. Zur Kultur und Praxis einer beweisenden Disziplin. (1. Aufl.).

[CR14] Hennen L (1992). Technisierung des Alltags. Ein handlungstheoretischer Beitrag zur Theorie technischer Vergesellschaftung..

[CR15] Knorr-Cetina K, Martinsen R (1995). Laborstudien. Der kultursoziologische Ansatz in der Wissenschaftsforschung. Das Auge der Wissenschaft. Zur Emergenz von Realität. (1. Aufl.).

[CR16] Konrad K (2004). Prägende Erwartungen. Szenarien als Schrittmacher der Technikentwicklung. (1. Aufl.).

[CR17] Latour B, Law J (1991). Technology is society made durable. A sociology of monsters. Essays on power, technology and domination.

[CR18] Latour B, Rheinberger H-J, Hagner M, Wahrig-Schmidt B (1997). Der Pedologenfaden von Boa Vista. Eine photo-philosophische Montage. Räume des Wissens. Repräsentation, Codierung, Spur. (1. Aufl.).

[CR19] Latour B (2005). Reassembling the social. An introduction to actor-network-theory. (1. Aufl.).

[CR20] Law J, Bijker WE, Hughes TP, Pinch TJ (1999). Technology, closure and heterogeneous engineering. The case of the Portuguese expansion. The social construction of technological systems. New directions in the sociology and history of technology. (7. Aufl.).

[CR21] Mack RL, Carroll JM (1995). Discussion. Scenarios as engines of design. Scenario-based design. Envisioning work and technology in systems development. (1. Aufl.).

[CR22] MacKenzie D, Wajcman J (2010). The social shaping of technology.

[CR23] Nardi BA, Carroll JM (1995). Some reflections on scenarios. Scenario-based design. Envisioning work and technology in systems development. (1. Aufl.).

[CR24] Ornetzeder M (2010). Sustainable technology: studies on user innovation, social learning and innovation networks.

[CR25] Pinch TJ, Bijker WE, Bijker WE, Hughes TP, Pinch TJ (1999). The social construction of facts and artifacts. Or how the sociology of science and the sociology of technology might benefit each other. The social construction of technological systems. New directions in the sociology and history of technology. (7. Aufl.).

[CR26] Popper KR (2005). Logik der Forschung.

[CR27] Raven RPJM, Heiskanen E, Lovio R, Hodson M, Brohmann B (2008). The contribution of local experiments and negotiation processes to field-level learning in emerging (niche) technologies. Meta-analysis of 27 new energy projects in Europe. Bull Sci Technol Soc.

[CR28] Rheinberger H-J, Rheinberger H-J, Hagner M, Wahrig-Schmidt B (1997). Von der Zelle zum Gen. Repräsentationen der Molekularbiologie. Räume des Wissens. Repräsentation, Codierung, Spur. (1. Aufl.).

[CR29] Rosson MB, Carroll JM, Jacko JA, Sears A (2003). Scenario-based design. The human-computer interaction handbook. Fundamentals, evolving technologies and emerging applications. (2. Aufl.).

[CR30] Schreuer A, Ornetzeder M, Rohracher H (2010). Negotiating the local embedding of socio-technical experiments. A case study in fuel cell technology. Technol Anal Strategic Manag.

[CR31] Schulz-Schaeffer I (2000). Sozialtheorie der Technik. (1. Aufl.).

[CR32] Schulz-Schaeffer I (2012). Scenarios as patterns of orientation in technology development and technology assessment.

[CR33] Star SL, Griesemer JR (1989). Institutional ecology. ‘Translations’ and boundary objects: amateurs and professionals in Berkeley’s Museum of vertebrate zoology, 1907–1939. Social Stud Sci.

[CR34] Strübing J (1997). Symbolischer Interaktionismus revisited. Konzepte für die Wissenschafts- und Technikforschung. Zeitschrift für Soziologie.

[CR35] Törpel B, Voss A, Hartswood M, Procter R, Voss A, Hartswood M, Procter R, Rouncefield M, Slack R, Büscher M (2009). Participatory design. Issues and approaches in dynamic constellations of use, design, and research. Configuring user-designer relations. Interdisciplinary perspectives. (1. Aufl.).

[CR36] van Eijndhoven J, van Est R, Joss S, Bellucci S (2002). The choice of participatory technology assessment methods. Participatory technology assessment. European perspectives. (1. Aufl.).

[CR37] Wellmer A (1967). Methodologie als Erkenntnistheorie. Zur Wissenschaftslehre Karl R. Poppers. (1. Aufl.).

[CR38] Winner L (1993). Upon opening the black box and finding it empty. Social constructivism and the philosophy of technology. Sci Technol Hum Values.

